# What Is ‘Missed Nursing Care’ During an Emerging Infectious Disease? A Concept Analysis

**DOI:** 10.1002/nop2.70216

**Published:** 2025-11-06

**Authors:** Mahsa Pourshaban, Hadi Hasankhani

**Affiliations:** ^1^ Department of Medical‐Surgical Nursing, Faculty of Nursing and Midwifery Tabriz University of Medical Sciences Tabriz Iran

**Keywords:** concept analysis, COVID‐19 pandemic, missed nursing care, nurse, nursing care

## Abstract

**Aim:**

Missed nursing care (MNC) is a global and important phenomenon in nursing and is universally used as an indicator of the quality of nursing care. However, no precise definition is available for this concept's dimensions and clinical features during an emerging infectious disease. This study aims to furnish a comprehensive evidence‐based definition of MNC in the context of the COVID‐19 pandemic.

**Design:**

A concept analysis paper.

**Methods:**

This study was conducted using an integrative approach to the concept analysis of Walker and Avant. In the literature review stage, the databases CINAHL, Web of Science, Scopus and PubMed as well as the Google Scholar search engine were searched from December 2019 to April 2024. Keywords of the study were selected according to the Medical Subject Headings (MeSH) and previous research. Textual analysis of the selected articles was conducted using an inductive and deductive approach. Throughout the study, the authors followed the SRQR checklist.

**Results:**

The results indicated the concept of ‘missed nursing care’ during an emerging and infectious disease such as COVID‐19 refers to a set of nursing activities and procedures that require interaction and close contact with patients and must be included in the care and treatment plan for patients (supportive, psychological‐social care and basic/bedside care). However, these activities have been presented as suboptimal, prioritised and interrupted. These attributes are caused by the complexity of caring for emerging diseases, aggravating lack of human and material resources, communication/teamwork and individual factors.

**Conclusion:**

The concept of MNC during an emerging infectious disease is an altered cognitive process that can be defined as disrupted nursing care (DNC) in the nurse role adjustment, time management and care environment for various reasons. COVID‐19 has been the most significant disruptor in healthcare, but it will not be the last.

**Implications for the Profession and Patient Care:**

This conceptual analysis can help sensitise care managers to the holistic view and adaptation of policies and strategies in crises, develop care models and theories, and help researchers generate specific tools or clinical scales for accreditation in emerging infectious diseases.

**Consent:**

No patient or public contribution.

## 1 Introduction

Missed nursing care (MNC) is a global and important phenomenon in nursing that refers to any aspect of patient care that is partially or completely omitted or delayed, and its occurrence is expected in all cultures and countries (Kalisch et al. 2009). In the last two decades, MNC has been observed under different titles such as ‘unfinished nursing care’, ‘implicitly rationed care’, ‘left undone’, ‘unmet nursing needs’ and ‘compromised nursing care (CNC)’ used in various texts (Bagnasco et al. 2020; T. L. Jones et al. 2015; Mandal et al. 2020; Palese et al. 2019; Recio‐Saucedo et al. 2018). Nevertheless, the term ‘missed nursing care’ is commonly used in the scientific literature to describe this phenomenon (Papastavrou and Suhonen 2021). MNC has attracted increasing interest from researchers, especially during the COVID‐19 pandemic, as some studies have compared missed care during this period with non‐pandemic periods (Monalisa et al. 2023). The international prevalence rate of MNC, based on nurses' reports of uncompleted necessary care activities, ranges from 55% to 98% (Kalisch et al. 2011). Studies have shown that MNC can lead to adverse patient outcomes such as pressure injuries, medication errors and infections, underscoring the importance of addressing this issue (Mandal and Seethalakshmi 2023). MNC can also have serious consequences, including decreased quality of care, decreased patient satisfaction, decreased nurse job satisfaction, increased patient complications, longer hospital stays and an increased likelihood of readmission (Chaboyer et al. 2021), and is considered a significant contributor to patient morbidity and mortality (Ball et al. 2014).

During the COVID‐19 pandemic, due to the highly dynamic clinical context and the implementation of previously unfamiliar guidelines, clinical nurses faced numerous challenges, such as excessive workload, physical exhaustion, role ambiguity, fear of infection, providing care with uncertainty, job burnout and moral distress, discomfort from of personal protective equipment (PPE), missed nursing care, prolonged procedures, lack of guidance during the outbreak, and lack of managerial support, which negatively affected nurses and the quality of patient care (Alsolami 2022; Cho and Steege 2021; LoGiudice and Bartos 2021; Stavropoulou et al. 2022). Patients with COVID‐19 have also experienced various physical and psychosocial challenges, such as panic and stigma, bad memories of being hospitalised (Firouzkouhi et al. 2023), shortness of breath, cough, fever and decreased consciousness. During this period, the common nursing diagnoses were hyperthermia, ineffective airway clearance, gas exchange disorder, self‐care deficiency, spontaneous ventilation disorder, spontaneous circulatory disorder, lack of knowledge and risk of shock (Hidayati et al. 2022). Additionally, the most common diagnoses according to NANDA nursing diagnoses were pressure injury risk, falls and infection. The diverse clinical manifestations of COVID‐19 encompass a wide range of both risk‐focused and problem‐focused nursing diagnoses and underscore the pivotal role of nurses in risk management (da Leenara Bezerra Silva et al. 2024). A survey conducted during the COVID‐19 pandemic revealed that 82% of patients did not receive a post‐discharge evaluation visit, and almost one in five reported unmet care needs (Healthwatch Report—Peoples Experiences of Leaving Hospital during COVID‐19 2020).

Concept analysis is thought to be particularly important in the development of nursing knowledge and theory (Meleis 2012). Concepts are generally recognised as central elements within theories. However, they have considerable theoretical significance in their own right. They allow for categorisation, organisation, labelling and discussion, thereby facilitating the study of phenomena within the discipline (Rodgers et al. 2018). In recent decades, various studies have reported the analysis of the concept of missed nursing care and its synonyms. The first analysis of this concept was presented in 2009 by Kalisch et al. (Kalisch et al. 2009). In one analysis, considered a healthcare rationing of nursing care was conceptualised as a deliberate decision to shortchange nurses in the performing their official duties to patients, which occurs due to the distribution of scarce resources (Moradi et al. 2023).

MNC is one of the concepts that has no boundaries for healthcare providers, especially nurses (Orique et al. 2017). Therefore, multiple interpretations can be made from manifestation. At the same time, this concept is complex and can have different meanings in different health care and crisis conditions (Albsoul et al. 2021), which requires clarification. Thus, considering that the concept analysis based on the Walker and Avant approach is used in the analysis of complex concepts that play an important role in the nursing profession, this approach was chosen to analyse this concept, as it provides logical and demonstrative framework to simplify an experimental concept (Walker and Avant 2019).

Despite the widespread attention given to MNCs, the key point of the present study is the lack of a clear definition of this concept in the unusual and adverse conditions created by the pandemic. Addressing this research gap is important in several ways. If we ignore the conceptual aspects of a phenomenon, we lose the ability to think and communicate clearly about it. A clear definition of this concept can lead to a common understanding among healthcare providers of the defining attributes, application, antecedents and consequences of MNC in the context of an emerging disease and help to understand the necessary differences in care during this acute and complex health challenge compared to normal conditions. Moreover, this understanding will facilitate regular monitoring, prediction, prevention, adaptation and change. As a result, the healthcare organization will be better prepared and more resilient in future crises to provide this gap can be valuable in the to patients and ultimately reducing mortality rates and healthcare costs. Addressing this gap can be valuable in the enhansing of nursing theory too. Given that the concept of MNC during the COVID‐19 pandemic is not clearly explained, the present study aims to examine this concept by defining its nature and the factors that influence its occurrence.

## 2 Methods

### 2.1 Study Design

The concept analysis was conducted according to the eight‐step framework of Walker and Avant: 1‐ concept selection, 2‐ determine the purpose of the analysis, 3‐ identify all applications of the concept, 4‐ choose the defining attributes, 5‐ construct a model case, 6‐ definition of additional items (borderline, related, contrary cases), 7‐ identify antecedents and consequences, 8‐ define the empirical references. the concept analysis based on the Walker and Avant approach is used to analyze complex concepts using logical and demonstrative approach to simplify an experimental concept (Walker and Avant 2019). The Merriam‐Webster medical dictionary and the Cambridge online dictionary were also used to define the concept of MNC during the pandemic.

### 2.2 Data Collection

The databases of CINAHL, Web of Science, Scopus and PubMed as well as the Google Scholar search engine were searched from December 2019 to April 2024. Study keywords were selected according to Medical Subject Headings (MeSH) and the previous studies' title, abstract and keywords section. The search process for this study included the use of the following search strategy in general and was done specifically for each database: ([‘omitted care’ OR ‘omission care’ OR ‘missed nursing care’ OR ‘unmet needs’ OR ‘left undone care’ OR ‘unfinished care’ OR ‘omitting care’ OR ‘implicitly rationed care’ OR implicitly care OR ‘unfulfilled care’ OR ‘forgetting care’ OR ‘neglecting care’ OR ‘elimination care’ OR ‘discontinuance care’] AND [Corona* OR COVID‐19*] AND nurse*). Boolean operators ‘AND’ and ‘OR’ and truncation functions appropriate for each database were used.

**FIGURE 1:**
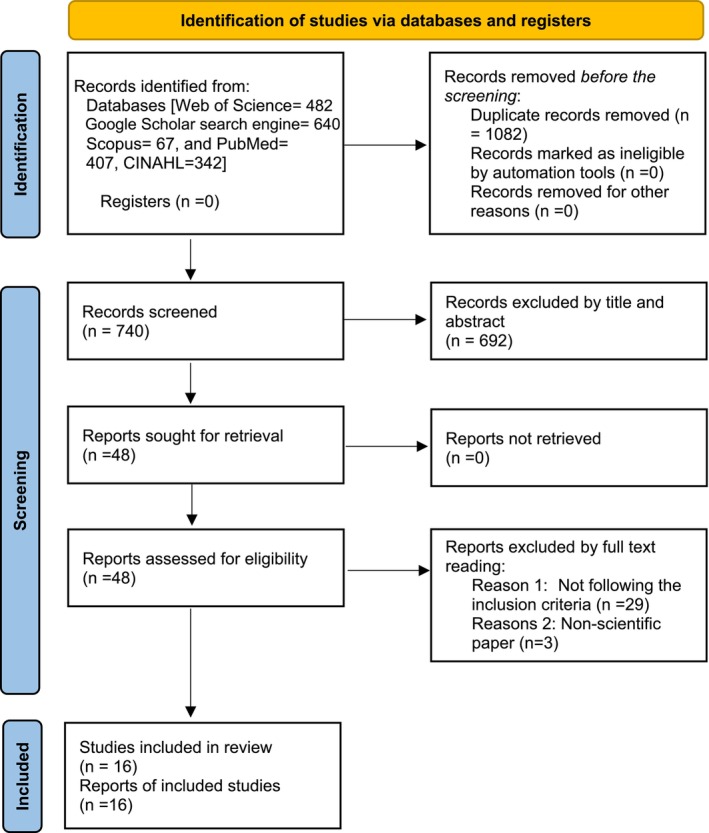
Process of paper selection: Diagram flow PRISMA.

Both quantitative and qualitative studies, the presence of relevant keywords in the title and abstract of the articles, and relevance to the concept of MNC in adult hospital wards during the COVID‐19 pandemic in all languages were included in the study. Articles unrelated to the topic of MNC during the COVID‐19 pandemic in adult wards and non‐scientific papers were excluded from the study. A total of 1938 studies were retrieved from online databases and stored using Mendeley software. One of the researchers reviewed the titles and abstracts of the selected articles. After removing duplicate (*n* = 1082) and irrelevant titles (*n* = 692), two researchers independently reviewed the full text of 48 articles. Thirty‐two studies were excluded for 2 reasons: 1‐ studies not following the inclusion criteria (*n* = 29), and 2‐ non‐scientific papers (*n* = 3). Sixteen related articles were finally selected for analysis. Figure 1 shows the search process that followed the guidelines of the Preferred Reporting Items for Systematic Reviews and Meta‐Analyses (PRISMA) search strategy (Page et al. 2021). Throughout the review, the authors followed the Standards for Reporting Qualitative Research (SRQR) checklist (O'Brien et al. 2014). Textual analysis of the selected articles was performed using an inductive and deductive approach. To ensure access to a sufficient number of studies in the field, the search process was conducted multiple times at different intervals.

This article is based on a specialised nursing doctoral thesis, approved with the code IR.TBZMED.REC.1401.1032 was conducted in 2023 to analyse the concept of MNC during the COVID‐19 crisis. The following were considered: careful selection of eligible studies, reporting of valid studies, conducting an impartial assessment of eligible studies, integrity and consistency in extracting results and avoiding any bias.

## 3 Results

### 3.1 Concept Selection

Despite developing similar concepts over the years and before the pandemic, the MNC concept was selected for analysis. This concept is commonly used in the scientific literature to describe this phenomenon (Papastavrou and Suhonen 2021). During the COVID‐19 pandemic, the biomedical perspective has predominated in these life‐threatening conditions due to the increased number of patients and limited healthcare resources in many countries. This can lead to an escalation of MNCs and a reduction in the quality of healthcare. The main challenge regarding this concept is the lack of a clear boundary for MNCs during this pandemic while having a clear definition of this concept in crisis can be useful for regular monitoring, prediction, prevention, adaptation and change. As a result, this clarity can enhance the preparednes and resilience of the healthcare organizations, enabling them to provide appropriate and safe care to patients while ultimately reducing mortality rates and healthcare costs.

### 3.2 Determine the Purpose of the Analysis

There are many different reasons for analysing a concept. It may be to examine the internal structures of a complex concept and identify its components, thereby increasing the exploratory power of the concept (Walker and Avant 2019). As MNC seems to be a fundamental concept in nursing (Imam et al. 2023), it is important to know how this concept is defined during the COVID‐19 pandemic crisis. By clarifying the concept of MNC during the COVID‐19 pandemic crisis, an understanding of how to differentiate this concept from others in adverse healthcare conditions can be provided to users of this concept and lead to concept development. The aim of analysing the concept of MNC during the COVID‐19 pandemic is to identify the applications of the concept, determine its defining attributes identify a model case, identify related, borderline and contrary cases, identify the antecedents and consequences of the concept, and define the empirical referents to reduce the ambiguity and semantic coherence of this concept in the context of an emerging infectious disease condition.

### 3.3 Identify all Applications of the Concept

Walker and Avant argue that providing an overall picture of the applications of the concept being studied provides a richer understanding of the concept and lends credibility to the attributes defined (Walker and Avant 2019).

#### 3.3.1 Dictionary Definition

No dictionary provides a single definition of missed nursing care. Merriam‐Webster's Medical Dictionary defines the word ‘care’ as the provision of treatment, attention and maintenance necessary to improve and maintain the health of patients. It refers to the process of providing health and medical care to patients, which includes diagnosis, treatment, prevention and continuing care to improve and maintain the health of patients. In this dictionary, the word ‘missed’ is defined as an adjective meaning ‘something that has been character or forgotten’. Generally, this word is used to refer to something that has been missed or forgotten and is no longer available (Merriam‐Webster 2021).

The Cambridge Online Dictionary defines the noun ‘care’ as ‘the process of protecting someone or something and providing what that person or thing needs. It generally refers to attention, concern and devotion to improving and maintaining the well‐being of an individual. In this dictionary, the adjective “missed” is defined as an’ opportunity or time that has passed and is no longer available. In general, it refers to something missed or lost because of a missed opportunity or chance (Cambridge Dictionary 2021).

Therefore, during the COVID‐19 pandemic, MNCs can be defined as interruptions and disturbances in providing care to improve and maintain patients' health and what the client/patient needs.

#### 3.3.2 Identification of Its Uses

MNCs can generally have a broad, multidimensional and complex range of meanings in different care contexts (Lopez‐Dicastillo et al. 2020). In one study, MNC was used as an index to assess the quality of care (C. Jones et al. 2019). To address this issue, some studies have included quality‐related questions alongside the MNC instruments to measure the quality of care during the COVID‐19 pandemic (Cengia et al. 2022; Falk et al. 2022; Labrague et al. 2022; Nymark et al. 2022; von Vogelsang et al. 2021). In another pre‐pandemic study, unfinished care predicted poorer quality of care, lower patient satisfaction, more adverse events, higher turnover, lower job and career satisfaction and higher intention to leave (T. L. Jones et al. 2015). Measuring MNC is also a strategy to increase patient safety (Palese et al. 2019). Under the conditions of the COVID‐19 pandemic, in addition to measuring MNC, some factors were considered and measured as predictors of this event. Factors such as the nursing work environment and its safety (Falk et al. 2022; Gurková et al. 2022; Khajoei et al. 2023; Labrague et al. 2022; Nymark et al. 2022; von Vogelsang et al. 2021), individual self‐understanding and identification of personal and professional feelings and strategies (Obregón‐Gutiérrez et al. 2022), satisfaction with the work environment (38), stress level (Mehrabian et al. 2023) and understanding of organisational support (Khajoei et al. 2023; Khrais et al. 2023).

The concept of MNC during the COVID‐19 crisis means that secondary, supportive and psychosocial care (Cengia et al. 2022; Gurková et al. 2022; Hosseini et al. 2023; Labrague et al. 2022; Mehrabian et al. 2023; Muhammadi et al. 2023; Obregón‐Gutiérrez et al. 2022; Safdari et al. 2022, 2023; Sugg et al. 2021) should be provided to the patient and missed or interrupted, and nurses should prioritise life‐saving care (Alfuqaha et al. 2023; Cengia et al. 2022; Falk et al. 2022; Gurková et al. 2022; Hosseini et al. 2023; Khrais et al. 2023; Labrague et al. 2022; Mehrabian et al. 2023; Muhammadi et al. 2023; Nymark et al. 2022; Obregón‐Gutiérrez et al. 2022; Safdari et al. 2023; Sugg et al. 2021). In other words, due to infection prevention measures and fear of contracting the virus, care that requires close contact and interaction with patients must be included in the care and treatment plan (ambulation, turning, oral care, wound care, education of patients and their families, and monitoring of the patient's condition) has been ignored (Alfuqaha et al. 2023; Cengia et al. 2022; Falk et al. 2022; Gurková et al. 2022; Hosseini et al. 2023; Khrais et al. 2023; Labrague et al. 2022; Mehrabian et al. 2023; Muhammadi et al. 2023; Nymark et al. 2022; Obregón‐Gutiérrez et al. 2022; Safdari et al. 2023; Sugg et al. 2021). These activities and procedures have been presented incompletely, inconsistently, inadequately or after a reasonable time has elapsed. In other words, MNC during the COVID‐19 pandemic refers to all priority nursing activities that nurses cannot perform completely or promptly for various reasons. As a result of being in a complex care environment and lack of time, the nurse makes a conscious decision to miss or postpone care so that in crises, in addition to self‐protection, the nurse can provide the necessary care to save the patient's life. MNC during a pandemic can lead to the spread of disease in the community and an increase in patient mortality. However, MNCs can be viewed less negatively in the context of a health crisis such as COVID‐19. When the existing risks outweigh the potential benefits, nurses' deliberate response to the challenges ahead can turn the threats posed by an epidemic into opportunities to keep patients and nurses as safe as possible (Lorentz et al. 2023).

During COVID‐19, MNC levels may remain the same or change slightly compared to before, what it was before, due to control of the care environment, such as adequate resources and equipment (Labrague et al. 2022), advanced preparedness to deal with the pandemic (Gurková et al. 2022), reducing the number of regular patients, and maintaining the nurse‐to‐patient ratio (von Vogelsang et al. 2021). The development of personal and professional strategies (Obregón‐Gutiérrez et al. 2022), proper performance of nurses with training programs (Khajoei et al. 2023), and more support from managers (Cengia et al. 2022) can be made possible.

#### 3.3.3 Relevant Concept

Based on the reviewed literature on MNC during the COVID‐19 pandemic, the definition of MNC is very close to the concepts of ‘rationing of nursing care’ (RONC) and ‘compromised nursing care’ (CNC) and it seems that these concepts can be used instead of the MNC concept during the COVID‐19 pandemic. Because RONC refers to nursing tasks that should be performed but that nurses refuse or fail to perform for reasons such as limited time, staffing or skill mix shortages (Schubert et al. 2007). RONC is the process of planned nursing decision‐making and prioritisation of incomplete care or limited access to care, which can lead to incomplete, delayed and missed care (Moradi et al. 2023). CNC refers to care that cannot be provided to the best of one's ability due to conditions such as lack of resources, equipment and personnel (Palese et al. 2019). With the increasing number of COVID‐19 patients and the need for care in hospitals, many nurses may not be able to provide the necessary care to their patients to the best of their ability due to conditions such as resource, equipment and personnel shortages (Zhang et al. 2021), and in this condition, healthcare professionals are stressed, overworked and financially unstable while caring for themselves, their families and their patients (Sethi et al. 2020).

The difference between the concept of MNCs during a pandemic and MNCs under normal circumstances lies in the uncertainty and more severe public health consequences that exist during a pandemic. In addition, the unfamiliar care environment and the complexity of care delivery pose a threat of greater deviation from standard procedures, increasing the likelihood of missed care and the stress on caregivers.

### 3.4 Choose the Defining Attributes

Defining attributes are features that are repeatedly mentioned in a concept and play a key role in distinguishing it from others (Walker and Avant 2019). The recurring defining attributes of MNCs during the COVID‐19 pandemic, as identified in the literature reviewed, do not operate in isolation and often overlap and interact. These attributes include 1‐ care environment attributes, 2‐ nursing role attributes and 3‐ time to care and untimely care. Each of these will be described below.

#### 3.4.1 Care Environment Attributes

The nature of the care environment can be considered as one of the external attributes for the loss of care. During the COVID‐19 pandemic, the dynamic, unfamiliar and unpredictable nature of the clinical context created problems for the provision of care (Hosseini et al. 2023; Mehrabian et al. 2023; Obregón‐Gutiérrez et al. 2022; Safdari et al. 2022, 2023; Sugg et al. 2021; von Vogelsang et al. 2021). In addition to the mentioned factors, the lack of necessary human and material resources and the organisation's support method (Khajoei et al. 2023; Khrais et al. 2023; Labrague et al. 2022) and management method (Cengia et al. 2022; Falk et al. 2022; Khrais et al. 2023; Safdari et al. 2022, 2023; von Vogelsang et al. 2021) in acute or more complex health challenges causes inadequate nursing services. It becomes more inappropriate and ineffective because the health care system is under significant pressure, and in this situation, a large number of patients need care. Therefore, nurses cannot provide optimal care to patients. As a result, patients may receive suboptimal and inappropriate care. Therefore, one of the attributes of missed nursing care during the COVID‐19 pandemic may be ‘suboptimal care’.

#### 3.4.2 Nurse Role Adjustment Attributes

During the COVID‐19 pandemic, the attributes of the role of the nurse can be found in a range of professional and personal attributes (Alfuqaha et al. 2023; Falk et al. 2022; Hosseini et al. 2023; Khajoei et al. 2023; Khrais et al. 2023; Labrague et al. 2022; Mehrabian et al. 2023; Muhammadi et al. 2023; Obregón‐Gutiérrez et al. 2022; Safdari et al. 2022, 2023; Sugg et al. 2021) and nurse perception (Alfuqaha et al. 2023; Cengia et al. 2022; Falk et al. 2022; Gurková et al. 2022; Hosseini et al. 2023; Khajoei et al. 2023; Nymark et al. 2022; von Vogelsang et al. 2021) to team communication to coordinate (Cengia et al. 2022; Khrais et al. 2023; Mehrabian et al. 2023; Muhammadi et al. 2023; Safdari et al. 2022, 2023) and communication with the patient to identify problems (Muhammadi et al. 2023; Safdari et al. 2022) and make decisions in the care process (Alfuqaha et al. 2023; Khrais et al. 2023; Safdari et al. 2022, 2023). Therefore, this attribute can be considered an internal and hidden attribute of MNC. Dangerous and unsafe working conditions can affect the way of providing care and decision‐making, and as a result, prioritise care and provide or not provide care. The nurse's decisions are also related to the individual characteristics and professional roles of the nurse (Papathanasiou et al. 2024). When the nurse's role is disturbed, some care is prioritised by the nurse, resulting in the loss of other care. This problem is the mechanism used by nurses to provide necessary care to patients in complex and difficult care conditions. Therefore, another feature of missed nursing care during the COVID‐19 pandemic is ‘prioritised care’.

#### 3.4.3 Time to Care and Untimely Care

The exacerbated shortage of nurses in dealing with the mass of patients infected with the Coronavirus is also evident in other emergencies and disasters. During the pandemic, the increase in the number of patients to the number of nurses (Alfuqaha et al. 2023; Cengia et al. 2022; Falk et al. 2022; Gurková et al. 2022; Hosseini et al. 2023; Khrais et al. 2023; Labrague et al. 2022; Mehrabian et al. 2023; Muhammadi et al. 2023; Nymark et al. 2022; Safdari et al. 2022, 2023; Sugg et al. 2021; von Vogelsang et al. 2021) led to a lack of time to provide the necessary care. On the other hand, the reduction of time spent at the patient's bedside to prevent the nurse from contracting the virus led to the elimination of some care (Hosseini et al. 2023; Safdari et al. 2022; Sugg et al. 2021). Defects in supplies and equipment (Khrais et al. 2023), the time‐consuming wearing of PPE (Hosseini et al. 2023; Safdari et al. 2023; Sugg et al. 2021), high demand for care and high workloads (Falk et al. 2022; Gurková et al. 2022; Hosseini et al. 2023; Khajoei et al. 2023; Khrais et al. 2023; Muhammadi et al. 2023; Nymark et al. 2022; Obregón‐Gutiérrez et al. 2022; Safdari et al. 2022, 2023; von Vogelsang et al. 2021) can also lead to interruptions in care and to some care not being provided or being provided at the wrong time. When there is a potential risk to the safety of the caregiver in the care environment, and when the human and material resources available to provide care conflict with the amount or timing of care to be provided, caregivers face interruptions in care. Therefore, another characteristic of care lost during the COVID‐19 pandemic is ‘interrupted care’.

### 3.5 Construct a Model Case

A model case is an example that should demonstrate all the defining attributes of the concept and be a pure example of it (Walker and Avant 2019). This model case exemplifies the defining attribute of MNC during the COVID‐19 pandemic crisis as previously described (suboptimal care, prioritised care, Interrupted care).

*Anna is a nurse who works in the COVID ward and tries her best to provide optimal care to the patients in her ward. Because one of her colleagues is infected with COVID‐19 and is on sick leave, there is a shortage of specialised nurses to care for the patients in the COVID ward, and Anna is responsible for caring for more patients than usual. In the evening shift, after administering medications and taking vital signs, Anna discharged two patients as ordered and admitted three new patients with COVID‐19 infections and in unstable conditions. She asks for help from the nursing supervisor, but the necessary support is not provided. One of the patients in bed number 6 has severe COVID‐19 symptoms and is using an oxygen mask, pulse oximeter, and IV lines. The patient calls for the nurse due to shortness of breath and requests assistance with repositioning. Due to fatigue and stress from the workload, Anna delegates this task to a nursing assistant and continues caring for the newly admitted patients without supervising the nursing assistant's care of the patient. The nursing assistant also has little desire to be at the patient's bedside due to the heat generated by the personal protective equipment, fear of infection, and lack of gloves and masks. After about an hour, Anna goes to bed number 6 to administer PRN medications, donning personal protective equipment, performing hand hygiene, and compensating for the drug shortage. The patient feels lonely and anxious due to isolation, limited visitation, and uncertainty about recovery, and expresses a need for more information about the disease and its treatment. Anna assists the patient in repositioning, but due to a lack of knowledge about the treatment process and time restrict, she is unable to properly educate the patient. Due to the high volume of work and the limitations in establishing non‐verbal communication and touch due to personal protective equipment, she seeks the help of a psychotherapist to help with the patient's mental state. However, the psychotherapist is not present in the hospital. While assessing the patient's dyspnea, Anna noticed that the oxygen machine was not working properly, but she was unsure if the dyspnea was solely due to the malfunctioning machine. Without taking any action to address the patient's emotional state, she leaves the room to seek help in resolving the technical problem with the oxygen machine*.

This case illustrates the key attributes of MNCs during the COVID‐19 pandemic.

### 3.6 Definition of Additional Items

Walker and Avant argue that defining attributes, which are the most salient features of the concept under study, can be difficult because they may overlap with some related concepts. Therefore, to help the researcher make judgements about defining characteristics, they suggest examining cases that are not the same as the concept of interest but rather are similar or opposite to it (Walker and Avant 2019). These cases include related, borderline and contrary cases.

#### 3.6.1 Borderline Cases

Borderline cases include some, but not all, of the defining attributes of a concept. Identifying boundary cases clarifies the essential requirements for a model case and reduces ambiguity at the boundaries between cases (Walker and Avant 2019). These attributes can be observed in the following example:

*Brian is a 28‐year‐old patient who is critically ill with COVID‐19 and is transferred to a specialized unit after a delay in diagnosis and referral. The number of nurses in this unit is insufficient for the number of patients. Sarah, a nurse working in this unit, is responsible for Brian's care. After Brian is transferred to the specialty unit, Sara administers Remdesivir as prescribed, but does not provide the patient with a full explanation of the drug and its side effects. Brian complains not only of shortness of breath and fever but also of headache and body aches. Sarah does not pay enough attention to Brian's symptoms and gives him pain relievers and fever reducers without explaining how these medications can help him recover*.

This is a case of poor care. Some, but not all, of the MNC attributes are present in this patient.

#### 3.6.2 Related Cases

Related cases that do not have the defined attributes of a concept but have similarities to the concept being analysed can lead to confusion and errors in concept definition (Walker and Avant 2019).

*Mr. A is a patient with advanced lung cancer who was admitted to the medical ward during the COVID‐19 pandemic. Due to swallowing difficulties, he has started receiving intravenous nutrition. However, he also requests oral nutrition. The patient's limited understanding of the disease and its treatment, has resulted in a disinterest in nursing education leading to feelings of isolation and withdrawal. Additionally, the painful nature of the treatments, has caused the patient to be uncooperative with the care plan, resulting in the unfortunate omission of some necessary nursing interventions*.

This case has none of the attributes of MNC during the COVID‐19 pandemic. However, because the patient was in the advanced stages of the disease and unable to feed orally, and due to feelings of despair and hopelessness, impaired perception and lack of cooperation, some necessary care was not received. This situation can be mistaken for MNC.

#### 3.6.3 Contrary Cases

Contrary cases refer to cases that do not have any defining attributes of the concept under study. These cases help to clarify and define the concept by presenting a clear difference from the concept under study (Walker and Avant 2019).

*Sina is a 46‐year‐old man admitted to the hospital's intensive care unit for COVID‐19. The patient‐to‐nurse ratio, equipment in the unit, and personal protective equipment are adequate. Sina is in critical condition and requires urgent medical and nursing care. His symptoms include fever, shortness of breath, muscle aches, and loss of appetite. Nurses immediately attend to Sina and initiate fluid therapy and medication. Oxygen is delivered through a ventilator to reduce the fever and pain, and antipyretic and analgesic medications are prescribed. Despite the need for isolation and intubation, the conditions were created for Sina to meet with his family while maintaining standard precautions. Nurses attend to his psychological needs and concerns and provide him with the necessary information. In addition to the social and psychological support provided by the nurses, a psychotherapist is present at Sina's bedside. The treatment team collaborates regularly. Nurses constantly monitor Sina's respiratory and mental status and provide timely bedside care, including hygiene, nutrition, and repositioning, using standard precautions. Information about his disease course, treatment, and care is fully documented*.

Therefore, in contrast to the proposed model case, there is clear evidence in this case of adequate and comprehensive timely care, with appropriate teamwork and sufficient care facilities. It lacks none of the defined attributes of the model. Therefore, the contrary concept of MNC would be timely, optimal, continued, and timely care.

### 3.7 Identifying Antecedents and Consequences

#### 3.7.1 Antecedents

Antecedents refer to activities, situations or events that occur before the concept and may lead to MNCs (Walker and Avant 2019). A literature review reveals several key factors of MNCs during the COVID‐19 pandemic crisis, which are discussed below.

- Complexity of emerging infectious disease care: The complexity of caring for an emerging infectious disease includes a lack of knowledge and information about the disease, a new situation and unfamiliarity with the disease, and uncertainty in care that leads to trial‐and‐error care. Conditions such as restrictions due to personal protective equipment (PPE) and reduction of time spent at the patient's bedside for fear of contracting SARS‐CoV‐2, changes in the work environment and the difficulty of working in isolation, rapid changes in information updates, high demand for care, arbitrary or forced withdrawal of care, Not providing care based on the nursing process, neglecting or forgetting to provide care, delays in providing care, restricting the presence of patient's companions/caregivers due to infection control, comorbidities, elderly patients, attrition from care, prolonging symptoms, challenging nurses and making patient care more complex compared to normal conditions (Hosseini et al. 2023; Khajoei et al. 2023; Mehrabian et al. 2023; Muhammadi et al. 2023; Nymark et al. 2022; Obregón‐Gutiérrez et al. 2022; Safdari et al. 2022, 2023; Sugg et al. 2021).
- Lack of human resources: The adequacy of nursing staff is one of the most important factors in patient care quality. Increased workload due to increased demand for care, urgent patient situations, unexpected increases in patient volume, and heavy admission and discharge activity during the COVID‐19 pandemic are some of the most critical factors of MNC due to inadequate nurse‐to‐patient ratio. Other factors include nurses' emotional or physical fatigue, overtime, experience and expertise, age, gender, fewer interprofessional team members such as psychotherapists, shift type and inappropriate prioritisation (Alfuqaha et al. 2023; Cengia et al. 2022; Falk et al. 2022; Gurková et al. 2022; Hosseini et al. 2023; Khajoei et al. 2023; Khrais et al. 2023; Labrague et al. 2022; Mehrabian et al. 2023; Muhammadi et al. 2023; Nymark et al. 2022; Obregón‐Gutiérrez et al. 2022; Safdari et al. 2022, 2023; Sugg et al. 2021; von Vogelsang et al. 2021).
- Lack of material resources: Lack of hospital resources and infrastructure, such as the unavailability of drugs and equipment relative to patient demand, malfunctioning equipment and tools, the level of hospital facilities and inadequate PPE, make it difficult to care for patients in crisis (Gurková et al. 2022; Khajoei et al. 2023; Khrais et al. 2023; Labrague et al. 2022; Mehrabian et al. 2023; Obregón‐Gutiérrez et al. 2022; Safdari et al. 2022, 2023; Sugg et al. 2021).
- Communication/teamwork failure: Inadequate team interactions and communication lead to weaknesses in interprofessional collaboration, lack of organisational support for team members, weak safety culture, weak supervision of nursing assistants' activities, lack of effective communication between nurses and patients, tension among team members, unsupportive and undesirable work environment, functional task‐oriented model of care, excessive documentation and duplication of records that can be considered in MNC during the pandemic crisis. Therefore, the likelihood of MNCs during the pandemic crisis increases without an effective teamwork culture (Cengia et al. 2022; Falk et al. 2022; Khajoei et al. 2023; Khrais et al. 2023; Labrague et al. 2022; Mehrabian et al. 2023; Muhammadi et al. 2023; Nymark et al. 2022; Safdari et al. 2022, 2023; von Vogelsang et al. 2021).
- Individual factors: Personal and professional strategies of nurses in caring play a significant role in the level of nursing care during the COVID‐19 pandemic. Also, the level of satisfaction with income, current professional situation, understanding of responsibility, nurses' perception of nursing fundamentals for quality care, perception of patient safety and knowledge of nursing staff competence are personal factors affecting MNCs during the pandemic crisis (Gurková et al. 2022; Hosseini et al. 2023; Khajoei et al. 2023; Khrais et al. 2023; Obregón‐Gutiérrez et al. 2022; von Vogelsang et al. 2021).

#### 3.7.2 Consequences

Consequences are events or outcomes that can occur after a concept or as a result of the concept (Walker and Avant 2019). The consequences of MNCs during the COVID‐19 pandemic crisis are extensive. These consequences form a continuum from partial damage at one end to catastrophic damage at the other. MNC results in the failure of nurses to provide complete and correct care (Labrague et al. 2022; Safdari et al. 2022), missing of some care (Cengia et al. 2022; Safdari et al. 2023), decreased quality of care (Alfuqaha et al. 2023; Gurková et al. 2022; Hosseini et al. 2023; Khrais et al. 2023; Labrague et al. 2022; Mehrabian et al. 2023; Muhammadi et al. 2023; Nymark et al. 2022; Obregón‐Gutiérrez et al. 2022; Safdari et al. 2023; Sugg et al. 2021; von Vogelsang et al. 2021), unmet basic patient needs (Falk et al. 2022) and decreased nurse and patient satisfaction (Hosseini et al. 2023). Implications for the future nursing workforce and work outcomes—mainly the challenge of retaining nurses and intentions to leave the profession (Gurková et al. 2022), decreased patient safety (Falk et al. 2022; Hosseini et al. 2023; Labrague et al. 2022; Muhammadi et al. 2023; Nymark et al. 2022; Safdari et al. 2023; Sugg et al. 2021; von Vogelsang et al. 2021), increased complications and mortality (Alfuqaha et al. 2023; Labrague et al. 2022). Table 1 summarises studies conducted on MNCs during the COVID‐19 pandemic between 2019 and 2024.

**TABLE 1:** Summary of studies conducted on missed nursing care (MNC) during the COVID‐19 pandemic between 2019 and 2024.

Author (s) years, country	Key findings	Attributes defining	Antecedents	Outcomes
Safdari et al. 2022, Iran	The increasing demand for care caused by the pandemic and the problems of the work environment have led to the failure of nurses to provide complete, correct care and sometimes to miss parts of care to patients	MNC in patient's emotional and psychological support, comfort/relaxation, patient Inability of nurses to provide complete and correct care Challenging care	Resource availability Communication failure Teamwork failure Adherence to the nursing process High workload Fear and fatigue of nurses	Failure of nurses to provide complete and correct care Loss of part of care
Safdari et al. 2023, Iran	Unpredictable, varying and stressful work environments, limited human and financial resources, and special characteristics of the disease and infected patients are major challenges for nurses in providing adequate, effective, efficient and timely care	MNC includes basic care needs, emotional and psychological support, nursing supervision Challenging care for patients, disease and organisation	1. Care‐related factors (Uncertainty in care, PPE‐related limitations, Attrition from care, Futile care) 2. Disease‐related factors (Extension of symptoms, Unpredictable peaks of the disease, Restriction on the presence of patients' companions) 3. Patient‐related factors (comorbidities, elderly patients and deterioration of patients) 4. Organisation‐related factors (Restrictions on equipment supply, Lack of staffing, Weakness in the team and inter‐professional cooperation, Unsupportive work environment)	Decreased quality of care Decreased patient safety
Sugg et al. 2021, England	Nurses caring for patients with SARS‐CoV‐2 faced challenges in supporting patients in all three care areas	MNC in communication and psychosocial care Any aspect of nursing care that is omitted or interrupted due to challenges posed by the SARS‐CoV‐2 virus Challenging care	High patient‐to‐nurse ratios Lack of nurse time Patient dependency Practice environment Significant workload, fatigue, anxiety and mental health symptoms among healthcare Wearing PPE	The safety and quality of patient care are at risk
Labrague et al. 2022, Philippines	MNC measure was low. Frontline nurses tended to miss clinical aspects of nursing care during the pandemic. In small hospitals, MNC is less reported. A more positive safety culture leads to less MNC	MNC in basic/bedside care and adequate patient surveillance	PPE adequacy Nurse staffing levels Patient safety culture Hospital facility size	A decline in the quality of patient care and safety, incomplete provision of care, failure to rescue patients and an increase in complications and mortality
Gurkova et al. 2022, Czech Republic	Improving the nursing work environment could be crucial in reducing missed nursing care	MNC in fundamental nursing care The nurse work environment (NWE) is a significant predictor of MNC, more so than individual nurse variables	Nurses' perception of nursing foundations for quality of care Satisfaction with their current position Overtime work Unfavourable work environments	Low‐quality care and inadequate care. Pandemics have resulted in: Implications for the future workforce and job outcomes for nurses—mainly the challenge of retaining nurses and intention to leave the profession
Falk et al. 2022, Sweden	Variations in specific care activities, reasons for missed care and workload differences compared to before the pandemic	MNC in basic care needs The impact of COVID‐19 on the workforce	Unavailable medications and supplies/equipment Overtime hours Inadequate number of staff Urgent patient situations and unbalanced patient assignments	Decreased patient safety and unmet basic patient needs
Nymark et al. 2022, Sweden	‐Rated patient safety significantly lower during the COVID‐19 pandemic ‐MNCs did not substantially increase during the pandemic	MNC in basic/bedside care Changes in nursing staff perceptions due to caring for a new patient	Unexpected rise in patient volume Urgent patient situations Inadequate number of staff Policies and guidelines were updated rapidly with daily changes	Unsafe and low‐quality care in individual and organisational dimensions
von Vogelsang et al. 2021, Sweden	The level of MNC, quality of care and patient safety was similar to before the pandemic with effective management by nursing managers	MNC includes interrupts in responding to patient needs	Unexpected rise in patient volume—Urgent patient situations—Inadequate number of staff—Unbalanced patient assignments	Impact on patient safety and quality of care
Hossini et al. 2022, Iran	Nurses reported common reasons for missed care, faced challenges due to increased workload and staffing issues and unfamiliarity with the disease significantly impacted care provision	MNC includes supportive and necessary care, bedside care and psychosocial care Challenging care	Faced challenges due to increased workload Staffing issues Unfamiliarity with the disease	A decline in quality and safety decreased the satisfaction of nurses and patients
Obregón‐Gutiérrez et al. 2022, Spain	Nurses were able to maintain and increase care quality during the COVID‐19 pandemic by developing personal and professional strategies	MNC in Psychosocial care Prioritised acute physical needs over psychosocial activities prioritising care	Lack of available labour and material resources Communication failure Personal psychological stress High care demand Physical barriers in the work environment Personal characteristics (age, experience and specialty)	Hurt to quality of care
Cengia et al. 2022, Italy	Nurses caring for COVID‐19 and non‐COVID‐19 patients did not show differences in the occurrence of unfinished care	MNC in basic/bedside care and emotional support Activities that nursing professionals were not able to complete due to various	Human resources factors Priority setting in human resources issues Communication failure	Loss of some aspects of care and care elements
Alfuqaha et al. 2023, Jordan	Missed nursing care increased during COVID‐19, with the inadequate number of staff nurses being the main reason	MNC in basic care Omission of any aspect of required patient care	Staff shortage Length of clinical experience Shift type Age	Reduced quality of services provided, increased patient mortality
Khrais et al. 2023, Jordan	Communication problems had the highest impact on missed nursing care Accountability and perceived organisational support were the most significant predictors of missed nursing care	MNC in basic care and interrupted in responding to patients' needs	Communication failure Higher patient Nurse ratio Years of experience Satisfaction with income Perception of accountability Organisational support Material resource	The overall decline in the quality of care
Mehrabian et al. 2023, Iran	69% of nurses reported missed nursing care occasionally, often or frequently	Omission or interrupted in providing essential nursing activities (attending interdisciplinary care conferences, basic care interventions and psychological support for patients)	Human and material resources Communication failure Rotating shift work Unexpected rise in patient Nursing occupational stress Experience in nursing	Decrease in the quality of patient care
Muhammadi et al. 2023, Iran	The most common missed nursing care in ICUs included controlling O2 saturation and recording patient information, hand washing and response to nurse calls, with the main causes being the lack of nursing staff and assistants, and the high volume of patient‐related activities	MNC in basic/bedside care and control and recording patient information MNC is defined as care that is needed by the patient but is missed or interrupted	Staffing shortages High workload Compulsory overtime Nurses' fatigue Communication failure	Impact on patient care quality and safety
Khajoei et al. 2023, Iran	The mean level of MNC was relatively low (52/5). Importance of negative pressure rooms	MNC due to the deficiencies in the work environment and the relationship of MNC with the demographic characteristics of nurses Challenging nursing care	Physical fatigue Overload work Employment status Type of ward Lack of psychological and emotional support Lack of financial support Age	N/A

Abbreviations: MNC: Missed Nursing Care; PPE: Personal Protective Equipment.

### 3.8 Define the Empirical Referents

According to Walker and Avant, concepts and their defining attributes are abstract, so they may not be good empirical indicators. They consider empirical referents to be recognisable features of concepts whose appearance is a sign of the concept's existence, and the purpose of defining them is to facilitate the measurement and identification of the concept and to help create research instruments (Walker and Avant 2019). Despite various studies on MNC during the COVID‐19 pandemic crisis, a literature review by the authors shows that there is no globally accepted measurement tool for assessing MNC during the COVID‐19 pandemic, and this issue has been considered a study limitation in some cross‐sectional studies (Alfuqaha et al. 2023; Cengia et al. 2022; Falk et al. 2022; Khajoei et al. 2023; von Vogelsang et al. 2021). In general, the instruments used to measure MNC during the COVID‐19 pandemic include general measurement tools and complementary and supporting instruments and items during this period.

#### 3.8.1 The General Instruments Used to Measure MNC During the COVID‐19 Pandemic Include

The 24‐item MISSCARE survey consists of three sections: a background section that includes questions on demographics such as age and gender, education level and experience in the role and current unit. There are also questions about workload: number of patients cared for on the last shift, number of hours usually worked, hours of overtime, number of shifts missed due to illness in the last three months, perception of adequate staffing, teamwork and whether they had any intention of leaving their current position. Section A consisted of 24 questions on elements of the MNC using a five‐point Likert scale. Section B included 17 questions on reasons for MNC with a four‐point Likert scale (Alfuqaha et al. 2023; Falk et al. 2022; Gurková et al. 2022; Hosseini et al. 2023; Mehrabian et al. 2023; Muhammadi et al. 2023; Nymark et al. 2022; von Vogelsang et al. 2021) This instrument has been translated, culturally adapted and psychometrically tested in some studies. The Missed Nursing Care Scale [MNCS] examines nursing care activities that were missed or omitted by nurses during their previous shift. The scale includes 12 nursing tasks divided into two domains, clinical care and planning/communication (Labrague et al. 2022). The Care Left Undone Scale is a consensus‐based nursing prioritisation tool from which two measures of care left undone are derived. First, the prevalence of any care left undone (binary measure). Secondly, a score indicating the amount of care left undone is calculated by summing the number of activities ticked per person (Obregón‐Gutiérrez et al. 2022). The Unfinished Nursing Care Survey is composed of two sections: Part A (elements of UNC) and Part B (reasons for UNC). The following six factors ere considered: communication, priority setting, nurses' aides' supervision, material resources, human resources and workflow predictability. Each factor was assessed using a 5‐point Likert scale, ranging from 1 (not a significant reason) to 5 (a very significant reason) (Cengia et al. 2022). In one study, two researcher‐made questionnaires were employed to assess the impact of the novel coronavirus disease (COVID‐19) on healthcare delivery and associated factors. The questionnaire on missed care included items on adherence to standard precautions, patient‐to‐patient and nurse‐to‐patient contact, and basic care. However, these questionnaires were not psychometrically evaluated (Khajoei et al. 2023). *The Causes of Care questionnaire* was designed by Blackman et al. (2014) in Australia. This 17‐question questionnaire includes three subscales of human resources, material resources and communication (Muhammadi et al. 2023).

#### 3.8.2 Additional, Complementary and Supporting Instruments and Items Used in the Studies Conducted During COVID‐19 Include the Following

The structured survey which was based on the *Fundamentals of Care model* included three sections and subsections in each of the areas of care on physical (hygiene, personal cleansing and toileting, eating and drinking, rest and sleep, mobility, patient comfort and patient safety, medication management), relational (establishing a relationship with patients, talking and listening, non‐verbal communication, shared decision‐making, communicating with relatives, carers and significant others) and psychosocial (dignity and respect, respecting patients' values and beliefs, wellbeing, anxiety and depression) (Sugg et al. 2021). *The Safety Climate Scale (SCS)*, is used to examine nurses' perceptions of their organisation's safety climate using a Likert‐type scale from 1 (disagree) to 5 (agree) (Labrague et al. 2022). *The Practice Environment Scale of the Nursing Work Index (PES‐NWI)*, comprises 31 items divided into five domains (two facility‐level domains: ‘nursing foundations for quality of care,’ ‘nurse participation in hospital affairs’; and three unit‐level domains: ‘staffing and resource adequacy,’ ‘nurse manager ability,’ ‘leadership, and support of nurses,’ ‘collegial nurse‐physician relations’) (Gurková et al. 2022). The *perceived organizational support* (POS) survey is used to evaluate the degree to which employees believe that their work organisation values their contribution and cares about their well‐being. The POS scale comprises 36 items, which are measured on a 7‐point Likert scale (0 to 6). A higher score indicates greater organisational support (Khrais et al. 2023). *Expanded nursing stress scale* (ENSS), (ENSS) is a tool utilised to assess the occupational stress experienced by nurses. The questionnaire comprises 57 stressful situations, which are divided into nine subscales: death, conflict with physicians, inadequate emotional preparation, problems with colleagues and supervisors, workload, uncertainty about treatment, patients, families and discrimination. Higher total scores indicate a higher level of occupational stress (Mehrabian et al. 2023). *A visual analog scale* is employed to assess the self‐perceived quality of care provided and the degree of autonomy. The scale ranges from 0 to 10. *Ad hoc questionnaire* was designed to collect data on sociodemographics, employment, skills and personal preoccupations related to the COVID‐19 pandemic. It also included a list of feelings that nurses have experienced while providing care during the pandemic, with a degree of agreement with the statements on a Likert scale from 1 to 10. Additionally, it included a list of *personal and professional strategies* that nurses have adopted to promote care (Obregón‐Gutiérrez et al. 2022). The *job satisfaction items* are assessed on a 5‐point Likert scale ranging from 5 ‘Very satisfied’ to 1 ‘Very dissatisfied.’ Absence levels, from 1 day to more than 6 days, and the plans of leaving the current position item (Alfuqaha et al. 2023).

The *quality of nursing care* is one of the most frequently used concepts in evaluating MNC. However, its multidimensional nature makes it difficult to define and measure, and its interpretation varies among nurses and patients, which poses a challenge in its analysis. To address this issue, some studies have included quality‐related questions alongside the MNC instruments to measure the quality of care (Cengia et al. 2022; Falk et al. 2022; Labrague et al. 2022; Nymark et al. 2022; von Vogelsang et al. 2021).

Consequently, the existing instruments for measuring MNC do not directly consider specific care situations such as pandemics and crises. This is mentioned as a limitation in several studies (Alfuqaha et al. 2023; Cengia et al. 2022; Falk et al. 2022; Khajoei et al. 2023; von Vogelsang et al. 2021), and the instruments lack items reflecting care for patients with infectious diseases. There may be other reasons for MNCs that are not covered in section B of the instrument, which could be a potential limitation during the evaluation of MNC during the COVID‐19 pandemic. Consequently, given that the credibility of the instrument is influenced by the care context, instruments that have high validity and reliability and specifically encompass the concept of MNC during the COVID‐19 pandemic should be developed to evaluate the type and factors affecting MNC during this period.

Ultimately, based on the analysis of the concept of missed nursing care in critical situations such as the COVID‐19 epidemic, it is recommended that a tool be developed using several items to evaluate the performance of nurses regarding care in the best way in these special periods. As a suggestion, the following items can be incorporated into the tool for measuring MNC during a pandemic crisis:

1. Infection prevention is a crucial aspect of healthcare. It is necessary to assess whether nurses adhere to the relevant health guidelines to protect themselves and patients from the risk of infection. These guidelines include the use of personal protective equipment such as masks, regular hand washing and the application of disinfectants to clean and disinfect surfaces, environments and medical equipment.
2. Education: It is important to assess whether nurses are attempting to enhance their understanding of the disease and provide patients with the requisite information regarding symptoms, treatments and prevention of COVID‐19. Furthermore, it is essential to evaluate whether they are communicating effectively with patients.
3. Intensified care: It is necessary to ascertain whether nurses provide more care and attention to patients with COVID‐19 than to other patients or vice versa.
4. Nursing equipment: It is necessary to ascertain whether the necessary equipment for the processing of the novel coronavirus is available and whether the nurses are using it correctly. Is personal protective equipment sufficient or not?
5. Personal and professional strategies: Evaluate whether nurses utilise personal and professional strategies to manage patients during the pandemic due to increased demand for care and changes in patient needs
6. Nurses fear that they and their families will be infected with the virus.
7. Level of the sensitivity of nurses to the death of patients and reducing the hope of their recovery (futile care).

In addition to incorporating these elements into the MISSCARE instrument, it is recommended that the degree of autonomy, job satisfaction, income, job environment satisfaction, job stress, understanding of patient safety and quality of care, understanding of responsibility, understanding of organisational support, absence levels, plans of leaving the current position, level of well‐being and mental status of nurses in terms of stress and anxiety be measured. Incorporating qualitative research methodologies, which entail measuring beliefs, attitudes, decision‐making processes and care priorities, can enrich the design of MNC tools. These items can facilitate a more comprehensive assessment of nurses' performance in critical situations and the development of special instruments for regular monitoring of MNC. Regular monitoring of MNCs can be employed for prediction, prevention, adaptation, change and resilience‐building in healthcare organisations. This, in turn, can facilitate the provision of appropriate and safe patient care, aiming to reduce mortality rates and healthcare costs.

### 3.9 Definition of the Concept

**FIGURE 2:**
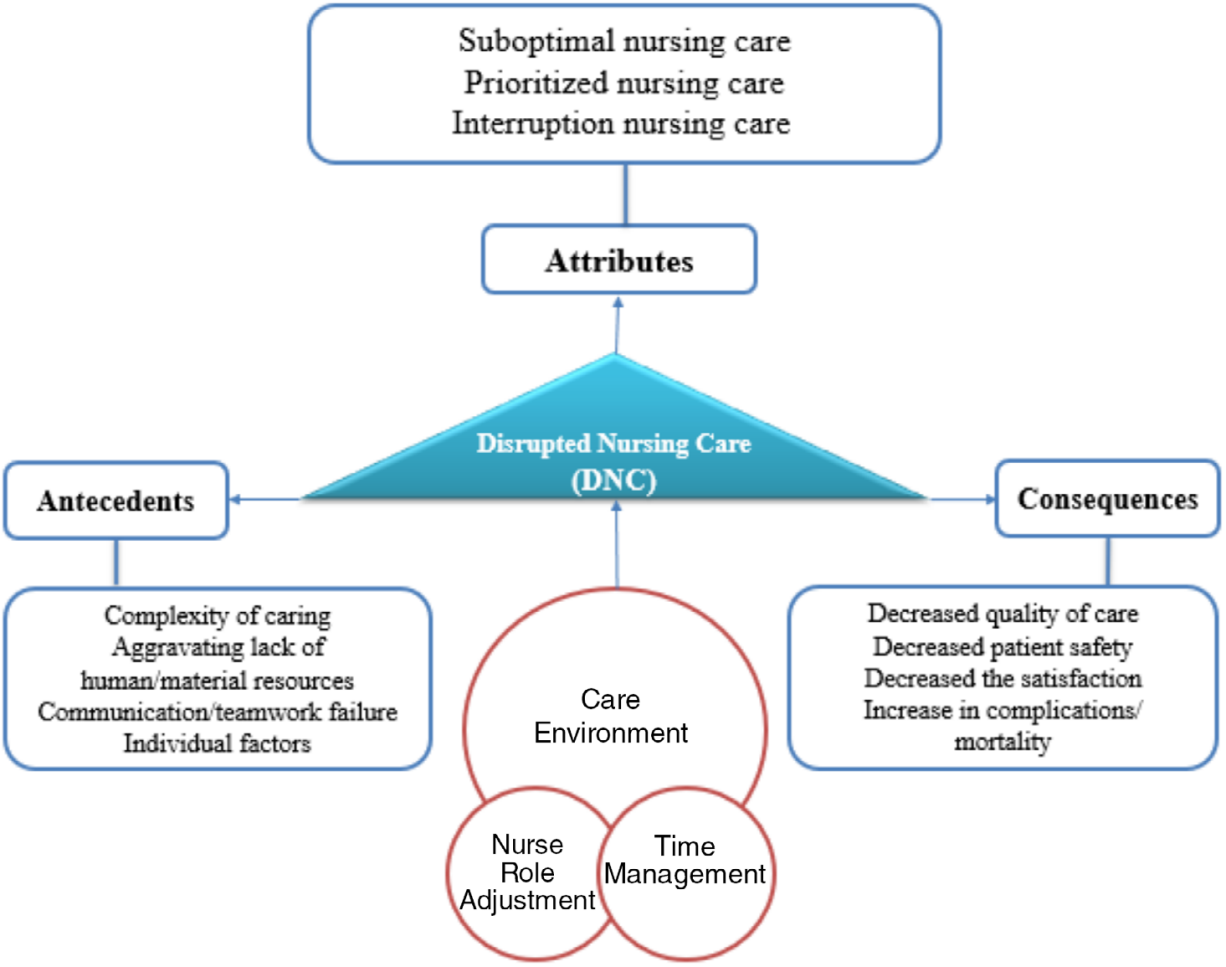
Antecedents, attributes and consequences of Disrupted Nursing Care (DNC) during the COVID‐19 pandemic.

Based on the present analysis the concept of ‘missed nursing care’ during an emerging and infectious disease such as COVID‐19 refers to a set of nursing activities and procedures that require interaction and close contact with patients and must be included in the care and treatment plan for patients (supportive, psychosocial and basic/bedside care). However, these activities were presented as suboptimal, prioritised and interrupted. These attributes are caused by the complexity of care for emerging diseases, aggravating lack of human and material resources, communication/teamwork failure and individual factors. In other words, this concept is an altered cognitive process and can be defined as Disrupted Nursing Care (DNC) in the nurse role adjustment, time management and care environment for various reasons. All of these have negative effects on the outcomes of patients. Figure 2 shows the conceptual model of MNC during COVID‐19 developed based on the findings of this study.

## 4 Discussion

This study used the Walker and Avant method was used to analyse the concept of missed nursing care for an emerging infectious disease. As a result of concept analysis through rigorous literature review, this concept is somewhat similar to previous definitions but differs in some aspects. The attributes of MNCs during the COVID‐19 pandemic are related to the intensification of limiting factors that pose challenges to providing care.

In this regard, studies have shown that during the COVID‐19 pandemic, three main issues arose that made it difficult to provide care. The first problem is related to the strength and capacity of health centres to respond (McGonigal 2020). The second is related to the change in behaviour and intensity of viral infection. This can cause the disease to spread rapidly and have the greatest impact on vulnerable and susceptible individuals (White et al. 2021). Finally, the paradoxical challenge of being close to and away from patients has reduced the amount of contact between patients and nurses. This may lead to a task‐oriented approach to nursing care and, in turn, may contribute to MNC (Jørgensen et al., 2022). Personal protective equipment was a challenge in therapeutic interactions with patients. Deprivation of family access to patients, increased workloads with increased patient admissions, the psychological distress of staff, and a decrease in the number of qualified nurses were also identified as problems during the pandemic (Digby et al. 2023). Also, the rate of nurses who had a negative experience was 96.9%. In addition, long working hours per shift and lack of training in public health emergencies made nurses more susceptible to work interruption (WI) (Zhou et al. 2021). The COVID‐19 pandemic has led to longer working hours for nurses, thereby increasing the risk of compromised care and suboptimal patient care (Senek et al., 2022). Changes in the work environment and staffing affect missed nursing care (Lake et al. 2020). The chaotic nature of the care environment creates challenging, unfavourable and unstable conditions, which in turn endangers the provision of comprehensive and safe nursing care. Therefore, the experience and preparation of nurses and managers to face such crises are very important. The nursing structure and environment in crisis should be designed and managed in such a way that it provides adequate nursing care to patients at the right time, and role‐playing a nurse should not be difficult.

The eligible studies showed that during the COVID‐19 pandemic, nurses prioritised life‐saving care and neglected or delayed secondary supportive and psychosocial care. In this matter, two issues are important which can be related to the time management, role adjustment, and environment care attributes. First, it is a conscious decision made by the nurse in response to time constraints and the complexity of the care environment (Moretti et al., 2021). When nurses have to make quick and independent decisions, they use intuition in clinical decision‐making (Aghajani et al. 2022). Second, moral sensitivity determines the context of decision‐making and clinical practice. It has an inverse relationship with the quality of nursing care, which during the pandemic was moderate (Darzi‐Ramandi et al., 2023). The results of one study showed that the prioritisation of care by nurses causes the cascading effect of missed care and occurs when care must be provided on time with limited resources, resulting in the postponement or elimination of some care tasks. As a result, a cognitive process is performed by nurses called stacking, which is a strategy to improve reprogramming in a macro‐cognitive work system (Patterson et al. 2011). Although this strategic mechanism of nurses can be considered positive, its consequences are more deviation from the standards of care, which were not very severe before an emerging disease and lead to suboptimal, prioritised and interrupted care. Also, the adverse consequences include a serious decrease in the quality of care and patient safety, and as a result, an increase in the death rate of patients.

Based on the analysis, multiple factors played a role in the occurrence of MNCs during the COVID‐19 pandemic, including the complexity of care in this era, aggravated lack of human and physical capital, communication failures and individual factors. In this regard, a review study identified five key issues affecting MNCs and noncompliance with infection prevention and control: organisation of care, staffing and resources; work environment; the context of care; managerial and interprofessional relationships; and individual nurse factors (McCauley et al. 2021). During the COVID‐19 pandemic, nurses faced many professional and psychological challenges. The main challenges include the payment system, management and supply of resources, psychological and moral distress, personal or family problems and motivational and welfare issues. (Jeleff et al. 2022). These relatively common factors and challenges, as well as the occurrence of nursing errors in different areas of care, can be a lesson for nurse managers who require their focused and special attention to correct and reduce their recurrence, thus improving the resilience of the health care organisation in health‐related crises. These relatively common factors and challenges can be lessons learned for nursing managers to improve and reduce the recurrence of MNCs.

The MNC during the pandemic was not solely attributable to the reluctance of nurses to provide care. Rather, certain aspects of the nurse‐patient care relationship have transformed in comparison to normal circumstances.

Stayt et al. (2022) refer to the concepts of ‘on a war footing’ and ‘doing the best we can’ in their discussion of care during the COVID‐19 pandemic (Stayt et al. 2022). The fragmentation of care and lack of a comprehensive approach led to inevitable consequences in care delivery, including suboptimal, prioritised and interrupted care. On the other hand, Kearns (2020) discusses the principle of ‘ought implies can’ regarding missed care (Kearns 2020). She states that if a nurse cannot provide a certain type of care, she is not morally responsible for failing if the reasons for the missed care are beyond the nurse's control. These philosophical perspectives can potentially enhance our understanding of developing coping mechanisms for MNCs in crisis. The salient fact is that this area of research is vast and requires further investigation.

## 5 Study Limitations

This is the first study to examine the MNC concept in a public health disaster, but this study has several limitations. First, the MNC concept was developed during the COVID‐19 pandemic based on existing literature. This is a methodological limitation. Therefore, conceptual analysis beyond literature‐based analysis is recommended. Second, the eligible studies were generally cross‐sectional. No reports were found outside of Europe and Asia, and this analysis may not reflect the global status of the concept, leading to a relative understanding of the concept. Third, there is a risk of subjectivity in the analysis of attributes and definitions. MNCs are often defined based on nurse judgement and standards of care, and the rates reported by MNCs may have been influenced. This is a constant limitation for most MNC studies. In addition, the instruments used in most studies did not have specific items for an infectious disease. What is standard under routine circumstances may not be considered standard during a disaster. Therefore, this issue emphasises the need for caution in interpreting and generalising findings.

## 6 Conclusion

Due to time constraints, a shortage of nursing staff, and limitations on care tasks, nurses are expected to face Disrupted Nursing Care (DNC). The clinical environment is a vital aspect of nursing practice, undergoing continuous transformation due to the challenges arising from the COVID‐19 pandemic. Nursing is a person‐centred, applied profession and missed nursing care in an emerging disease can be considered as a disruption in the cognitive and environmental process disruption. Nurses are the assets of healthcare organisations, and their decisions to perform specific nursing activities are influenced by their perceptions. Internal perceptions include team norms, decision‐making styles, values, beliefs and attitudes by which nurses understand their roles and responsibilities. In a pandemic, where the nurse's health is at risk while providing care and the care environment is highly variable and chaotic, the nurse, in fulfilling her/his role, provides care in a disrupted, suboptimal, prioritised and interrupted manner. The nurse makes a conscious decision about what care to provide, what care to delay or what care to omit to not only protect herself but also to provide the care necessary to save the patient's life. In other words, for various reasons, the nurse is faced with a disruption in role adjustment, time management and control of the care environment, and ultimately DNC occurs, which differs from previous terms of this concept.

The distinction between MNC and DNC is based on the assumption that MNC is an unconscious decision, whereas DNC is a deliberate and conscious decision. This distinction is made in response to the high‐risk care environment and subsequent role disruption, as well as the time required for care and the time to provide care. The presence of environmental threats during the COVID‐19 pandemic, combined with resource scarcity, distinguishes this situation from RONC.

Because healthcare workers are at risk during an infectious disease outbreak, patient care requires special support from nurses. A well‐equipped and safe work environment and support from the organisation will result in nurse safety, time management and more optimal decision‐making. This makes it easier to play the role of a nurse. The sum of these cases will lead to more optimal and safer patient care, and the result will be a reduction in mortality rates, a reduction in the spread of disease, a reduction in the duration of hospitalisation, and a reduction in the cost of healthcare services. COVID‐19 has been the most significant disruptor in healthcare, but it will not be the last.

## 7 Implications for Practice, Policy, Theory and Research

This conceptual analysis can contribute to the sensitivity of care managers towards the holistic view and the revising and adaptation of policies and strategies according to the crises. It can be used to develop care models and theories. Since MNC is an indicator to measure the safety and quality of care, the current conceptual analysis can help researchers generate specific tools or clinical scales for accreditation in emerging infectious diseases. The result of regular monitoring of MNCs can be prediction, prevention, adaptation and change, and as a result, the resilience of the healthcare organisation.

## Author Contributions

Mahsa Pourshaban Oushibi: investigation, methodology, validation, data extraction, interpretation of data, writing – original draft, formal analysis, review of the selected articles. Hadi Hasankhani: writing – review and editing, supervision, validation, data extraction, conceptualisation, review of the selected articles.

## Ethics Statement

This article is based on a specialised nursing doctoral thesis that has been approved with the code IR.TBZMED.REC.1401.1032 and financially supported by the Vice‐Chancellor for Research and Technology of Tabriz University of Medical Sciences.

## Consent

No patient or public contribution.

## Conflicts of Interest

The authors declare no conflicts of interest.

## Data Availability

The data that support the findings of this study is available from the corresponding author upon reasonable request.
